# Stabilizing patterns in time: Neural network approach

**DOI:** 10.1371/journal.pcbi.1005861

**Published:** 2017-12-12

**Authors:** Nadav Ben-Shushan, Misha Tsodyks

**Affiliations:** 1 Department of Physics, The Weizmann Institute of science, Rehovot, Israel; 2 Department of Neurobiology, The Weizmann Institute of science, Rehovot, Israel; University of Pittsburgh, UNITED STATES

## Abstract

Recurrent and feedback networks are capable of holding dynamic memories. Nonetheless, training a network for that task is challenging. In order to do so, one should face non-linear propagation of errors in the system. Small deviations from the desired dynamics due to error or inherent noise might have a dramatic effect in the future. A method to cope with these difficulties is thus needed. In this work we focus on recurrent networks with linear activation functions and binary output unit. We characterize its ability to reproduce a temporal sequence of actions over its output unit. We suggest casting the temporal learning problem to a perceptron problem. In the discrete case a finite margin appears, providing the network, to some extent, robustness to noise, for which it performs perfectly (i.e. producing a desired sequence for an arbitrary number of cycles flawlessly). In the continuous case the margin approaches zero when the output unit changes its state, hence the network is only able to reproduce the sequence with slight jitters. Numerical simulation suggest that in the discrete time case, the longest sequence that can be learned scales, at best, as square root of the network size. A dramatic effect occurs when learning several short sequences in parallel, that is, their total length substantially exceeds the length of the longest single sequence the network can learn. This model easily generalizes to an arbitrary number of output units, which boost its performance. This effect is demonstrated by considering two practical examples for sequence learning. This work suggests a way to overcome stability problems for training recurrent networks and further quantifies the performance of a network under the specific learning scheme.

## Author summary

The ability to learn and execute actions in fine temporal resolution is crucial, as many of our day to day actions require such temporal ordering (e.g. limb movement and speech). Indeed, generating stable time-varying outputs, using neural networks has attracted a lot of attention over the last years. One of the core problems, when facing such a task, is the solution stability, hence it was only possible to produce the sequence for a limited number of cycles. Here we propose a robust approach for the task of learning time-varying sequences.

## Introduction

There are many human behaviors which unfold over time. Our limb movement, speech and even our internal train of thought appear to involve sequences of events that follow one another in time. We are capable of performing an enormous number of sequences, and we can perform the same action in a variety of different contexts. Hence the concept of generating temporal patterns or sequences by neural networks draw a lot of attention over the years. Early work relied on cyclic inhibition [1–3] which formed the basis of networks that function as ring oscillators [4]. These models could only be applied to small number of neurons and are restricted in the complexity of the output they can generate. The complexity of a sequence is determined by the number of actions that must be remembered in order to know to correct successor. Later work [5, 6] produced temporal sequences in an arbitrary large network, using associative neural network with Hebb learning rule [7], encompassing the relation between output pattern and synaptic connections. The main idea in this model was to functionally separate the synaptic connection into two components, slow and fast, such that the slow component encoded transition between patterns and the fast component stabilized the current pattern. This model, in its basic form, only encodes transitions between neighboring states in a sequence. Hence it is also limited in the complexity of outputs it can produce. Specifically, in order to learn two partially overlapping sequences one should introduce another component in the synaptic connection, with time scale proportional to the amount of overlap between the sequences. Jordan first considered a clear distinction between the state of the network and the output [8]. Moreover, applying recurrent links within the network, provides it a dynamic memory by which “time” is implicitly encoded in the state of the network [9]. This kind of network architecture (i.e. recurrent and feedback connections) is common in cortical microcircuit [10, 11], hence various training schemes for such network architectures arose along the years. A generalization of this approach considered reading out target information from randomly connected network, was first suggested in [12] and later developed to the notion of echo state networks (ESN) [13] and liquid state machines (LSM) [14]. Typically these networks consists of non-linear activation function for units within the network “reservoir” which linearly combines the output signal. These models do not need an internal pacemaker for producing a temporal sequence, in addition, learning a complex sequence is deduced to effectively learning a simple sequence, as two highly overlapping sequences end up as distinct in the high dimensional phase space of the network. None the less, it has been found as a challenging task to establish a successful learning procedure for these networks, one in which the network is capable of reproducing a desired target sequence for an arbitrary number of cycles, yet exhibiting robustness to errors and noise which are assumed to be common in biological networks. The main difficulties in this context are: In order to achieve a stable solution one should use a long training period involving noise over the output unit. During training the network will sample various fluctuations which improves the final network stability [15]. The second difficulty is assigning credit to output errors, i.e. which neurons and synapses are most responsible for the output error. Previous work settled this issue by restricting modification to synapses which project directly to the output unit [16]. This assumption was supported by [17], in which they showed that even in the case that all synapses were subject to modification during training, the synapses to the output tended to change the most.

In our model we suggest a variation of the ESN, i.e. For a recurrent network with a feedback loop, we consider linear activation function for neurons within the network and a binary output unit. In such case, given a target sequence on the output unit, one may easily solve for the corresponding activity in the network. Following previous work [16] we restrict ourselves on modifying synapses which project directly to the output unit. Even though it causes the solution space to shrink, it makes the learning problem straight forward, as it can be reduced for solving a simple perceptron [18, 19] problem.

This approach settles the problem of feeding back erroneous output to the network. Robustness to errors and noise naturally emerges from the finite margin of the perceptron problem, thus reproduction of a target sequence for an arbitrary number of cycles is possible, even in the presence of noise. In addition, considering a binary output unit helps in better quantifying the network performance, hence providing a different view on the computational power of this class of networks.

In our model, quantifying the memory capacity (MC) of the network is mathematically equivalent to calculating the capacity of a perceptron with correlated patterns presented to it. Where correlation induced by the network dynamic, as such, this is a challenging task analytically. Similar problems had been tackled in [20, 21] for the simplified case in which each neuron maintained an activity trace consisting of a decaying sum over all previous inputs presented to it. In [22] they considered correlated input-output associations, where temporal correlations between binary input patterns were modeled as Markov chain. In this case analytic result could only be obtained for the case of no temporal correlation between input patterns. Both models form a simple feed-forward architecture, hence temporal correlation do not depend on the state of other neurons in the network. In our model temporal correlation are of higher complexity due to the recurrent connectivity, hence we use numeric simulations in order to quantify the memory capacity for both the discrete and continuous time cases. Specifically, we solved the soft margin perceptron problem (Methods) with *matlab* standard quadratic programming function. An analytic estimation is given to noise robustness of the system.

## Results

### Discrete time case

#### Network model

**Fig 1:**
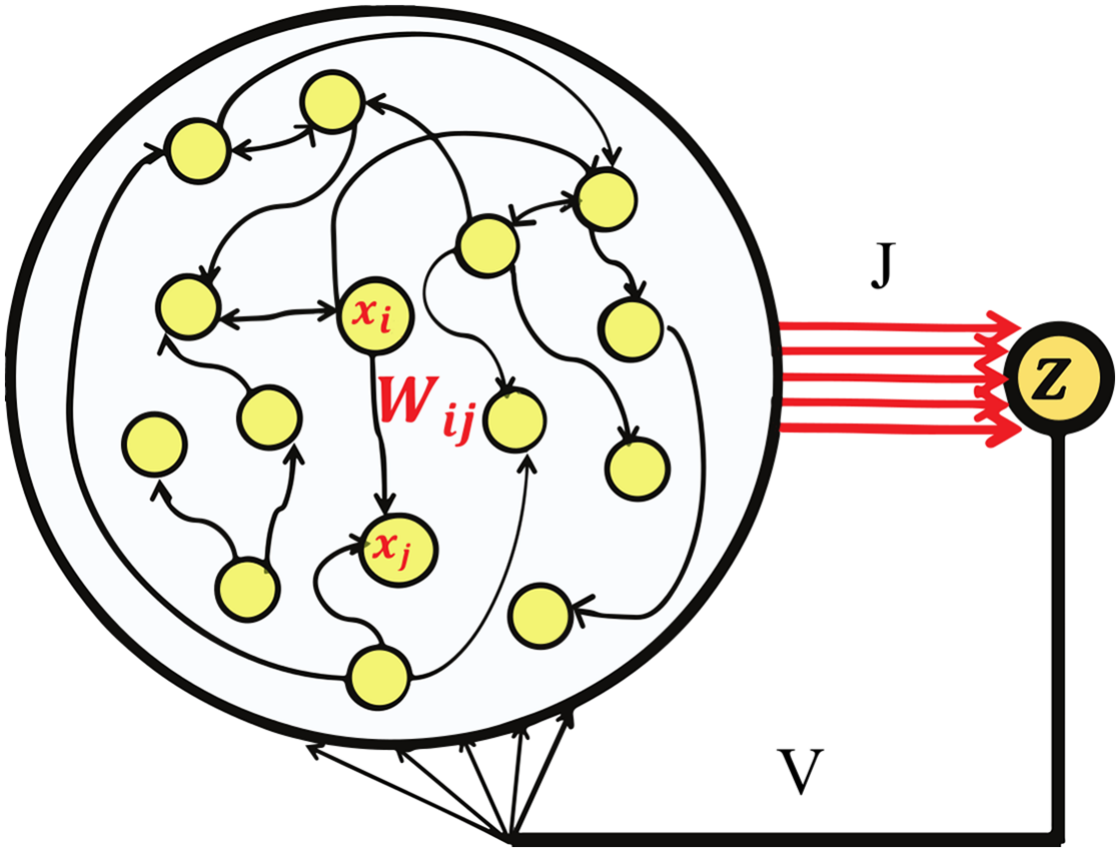
Network architecture. The *N* generator neurons, **x**(*t*), displayed in the large circle, are connected within themselves randomly, connections are represented by matrix **W**. In the figure *W*_*ij*_ is the strength of connection from neuron *i* to *j*. The generator neurons are connected to the output unit *z*(*t*) via the weight vector **J**. The output unit is recurrently connected to the generator neurons with weight vector **V**. During simulation we will only modify the output weights, **J**, and leave **W** and **V** constant.

We use a recurrent and feedback neural network architecture (Fig 1). We start by analyzing the simpler, discrete time case with the following dynamic equations:

$$\begin{array}{ccc} x(n+1) & = & Wx(n)+Vz(n)+\eta (n) \end{array}$$ (1)

$$\begin{array}{ccc} z(n) & = & \text{sign}(J\cdot x(n)) \end{array}$$ (2)

Where **x**(*n*) represent the activity pattern in the *n*^*th*^ time step over the pool of *N* generator neurons. These are randomly connected within themselves, the matrix **W**, represent these random connections. The vector **J** stand for the synaptic weights from the network to the binary output unit *z*(*n*), **V** stands for the synaptic weights of the feedback loop, i.e. from the output unit back to the network. We also included an uncorrelated random noise term, ***η***(*n*), with the following statistics, 〈*η*_*i*_(*n*)〉_*n*_ = 0 and ${\langle \eta_{i}\left(n\right)\eta_{j}\left(n+k\right)\rangle }_{n}=\sigma_{noise}^{2}\delta_{ij}\delta_{k0}$, where 〈⋯〉_*n*_ denotes a time average.

The random connections within the network are drawn from $W_{ij}∼N\left(0,\frac{\lambda^{2}}{N}\right)$, note that by choosing λ < 1, we force the largest eigenvalue of **W** to be smaller than one (in absolute value) [23]. This choice ensures that the entire dynamic is restricted to stable manifolds. The synaptic weights in the feedback loop are random as well and drawn from a normal distribution, later we apply the normalization ‖**V**‖ = 1, to scale with our choice of **W**. Our goal is thus to find an appropriate set for the output weights, **J**, which are capable of holding a desired set of dynamic memories. Specifically we are interested in learning periodic sequences, motivated by the periodic nature of many motor actions (e.g. running, swimming or bouncing a ball). As such we would like the network to be capable of reproducing a desired sequence for an arbitrary number of cycles.

Given a specific target sequence ${\left\{z_{t}\left(n\right)\right\}}_{n=0}^{T}\equiv \left\{z_{t}\left(0\right),..,z_{t}\left(n\right),..,z_{t}\left(T\right)\right\}$, one can use Eq (1) to solve recursively for the activation pattern over **x** neurons in each time step:

$$\begin{array}{c} x\left(n\right)=W^{n}x\left(0\right)+\sum_{k=0}^{n-1}W^{k}Vz_{t}\left(n-k\right) \end{array}$$ (3)

where we omitted the noise term while deriving Eq (3), since we are interested in the trajectory in phase space induced by a given target sequence, i.e. this is the exact trajectory we would like the network to follow. Demanding that the network will reproduce the sequence periodically we set **x**(*T*) = **x**(0), thus finding the appropriate initial condition, **x**(0) ≡ **x**_0_:

$$\begin{array}{c} x_{0}={\left(I-W^{T}\right)}^{-1}\sum_{k=1}^{T-1}W^{k}Vz_{t}\left(T-k\right) \end{array}$$ (4)

Eqs (3) and (4) uniquely defines the generator neurons activity, or target activity for a given target sequence at each time step. Hence we have a set of patterns and their labels ${\left\{x\left(n\right),z_{t}\left(n\right)\right\}}_{n=0}^{T-1}$. We note that using the simple procedure described so far, we are able to cast the temporal learning problem to a simple perceptron problem. That is, we need to find an appropriate set of output weights, **J**, that classify correctly the training set, i.e. satisfies Eq (5) at each time step:

$$\begin{array}{c} z_{t}\left(n\right)=\text{sign}\left(J\cdot x\left(n\right)\right) \end{array}$$ (5)

If there exists a solution for the perceptron problem, cuing the network with the appropriate initial condition, **x**_0_, will cause the network to reproduce the desired target sequence over and over again, as by construction it is a periodic orbit.

#### Network performance

**Fig 2:**
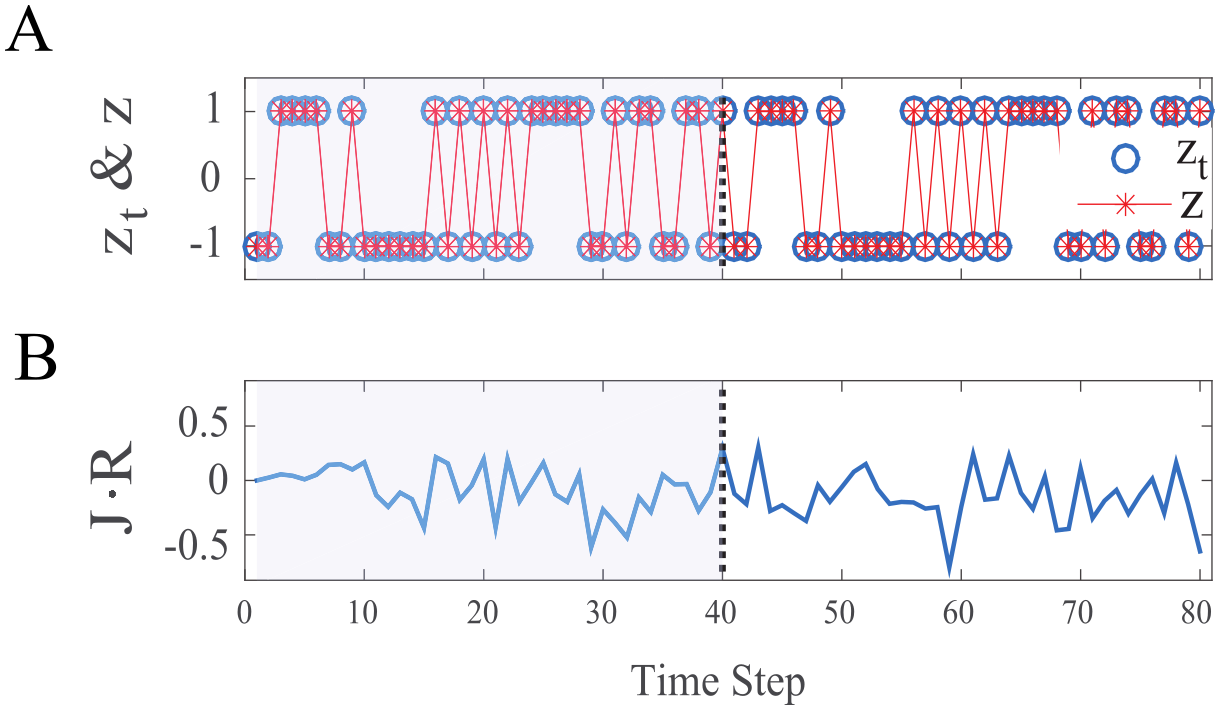
Learning a single sequence. We used network of size *N* = 100, with λ = 0.99 normalization to learn a 40 time step target sequence. After the learning procedure we cued the network with the proper initial condition and let it naturally evolve by Eqs (1) and (2) with $\sigma_{noise}^{2}=10^{-2}$ (where the network activity is O(1)). In order to emphasize the model robustness, we show 2 cycles of the network dynamic. Dashed black line indicates the end of the first cycle. Generally Blue colors are used for the desired activity in the network and Red colors for the network activity after the learning procedure (A) The target sequence and the network output after learning, the network produces the exact target sequence with no errors (B) The projected error, **R** is the difference between the noiseless target activity to the noisy dynamics after learning. Note that noise driven deviations are kept small, indicating the solution is robust.

In the following (Fig 2) we show a demonstration of our suggested learning procedure results. We used an *N* = 100 network with λ = 0.99 normalization in order to learn a desired target sequence of 40 time steps. After inferring the proper weights by our learning procedure we let the network naturally evolve with Eqs (1) and (2) and $\sigma_{noise}^{2}=10^{-2}$, where the network activity is O(1). For clarity we only show 2 cycles of the network dynamics, in which we notice the perfect performance of the network, despite the finite noise. We note that as expected the learned periodic orbit is stable, as small perturbations driven by noise, exponentially decay. Let us now turn on quantifying the performance of our system.

**Fig 3:**
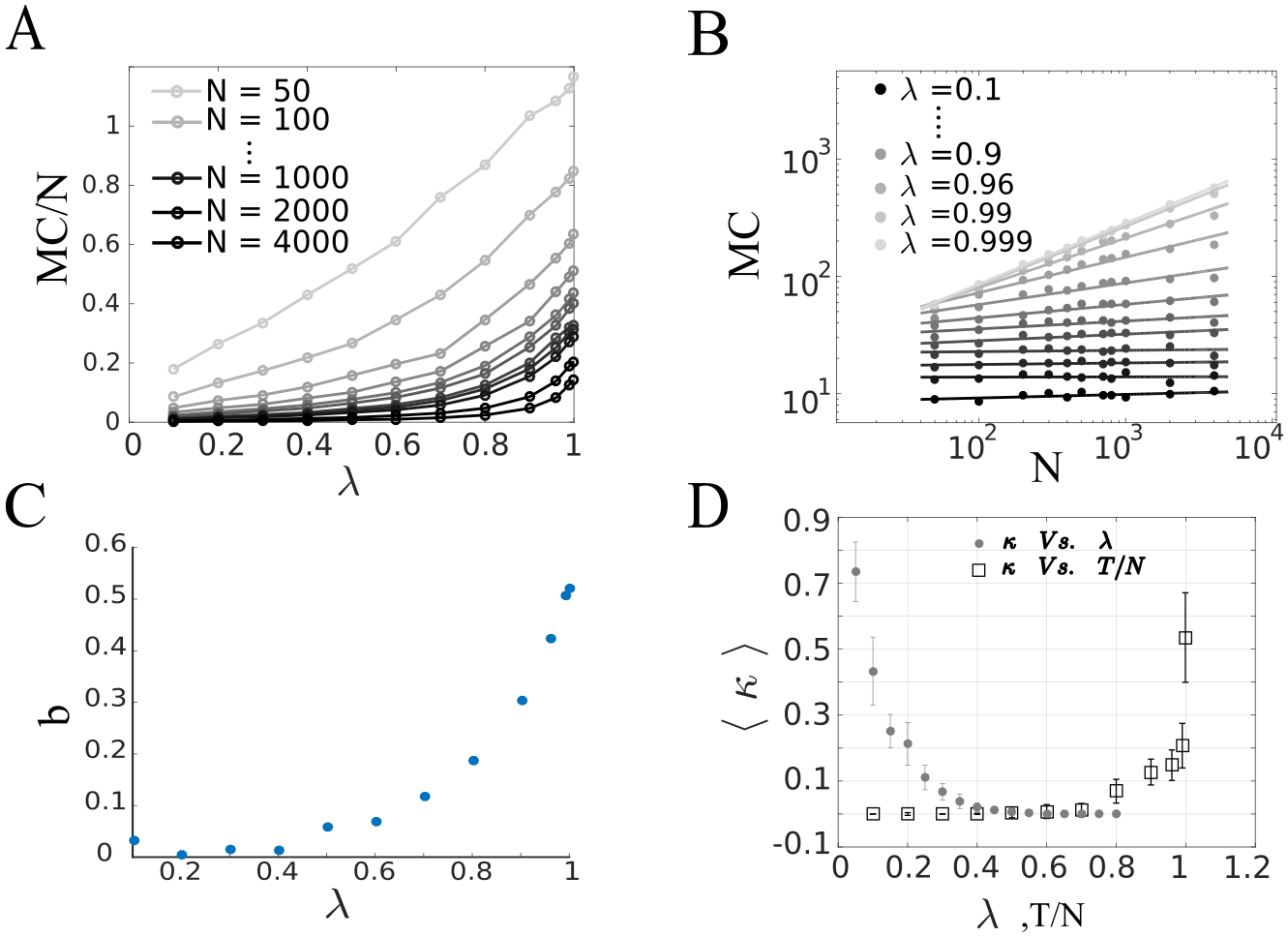
The memory capacity for a single sequence. (A) The memory capacity normalized by the network size as function of λ—the largest eigenvalue of the connectivity matrix **W** in absolute value. The MC monotonically increase as we increase λ. Note that by increasing λ, we increase the number of eigenvectors with long decay times. On the other hand, the MC does not seem to scale with network size, *N*, but rather sub-linear with it. (B) probing the scaling of the MC with the network size. In the log log plot, the MC seems to linearly scale with the network size. But with different slope, *b*(λ), for every λ. Filled circles are simulations results, solid lines are least squares fit to these points (C) scaling of the exponent, *b*, as function of λ. For λ → 1^−^, $MC∼\sqrt{N}$. For small values of λ it seems that the MC saturates, *b* ≈ 0 (D) the solution margin, for a fixed sequence length, monotonically increase as we increase λ. On the other hand, the solution margin monotonically decreases as we increase the sequence length, for λ = 0.999.

*The longest single sequence.* As a first step in quantifying the network performance we ask *what is the longest single sequence that the network is capable of learning?*. The length of the longest sequence defines the memory capacity (MC) of the network. Since we are in a discrete time case we measure it in time steps. We examine how the MC varies with respect to the parameter λ—the largest eigenvalue of **W** in absolute value (See Methods). The target sequence, {*z*_*t*_}, is binary such that *z*_*t*_(*n*) = ±1 with probability ½. The memory capacity for a given network size, *N*, and specific normalization parameter λ, is the longest sequence the network can learn, such that on average (over many different target sequences) the network can reproduce a target sequence without a single erroneous bit. Simulations suggest (Fig 3), that for a given network size, *N*, The memory capacity increases as we increase λ. This is an intuitive result, as increasing λ also increases the effective decay time of the network. Indeed the ability to maintain ongoing activity in the network for longer time is intimately related to the memory capacity as simulations suggest. In addition, for a fixed sequence length, increasing λ, increases, on average, the solution margin κ (Fig 3D). The MC relation to the network size seems to be λ dependent, and generally behave as a power law, *MC* ∼ *N*^*b*(λ)^ (Fig 3C). For λ → 1^−^, the MC scales roughly as $\sqrt{N}$, and for small values of λ the MC seems to saturate.

**Fig 4:**
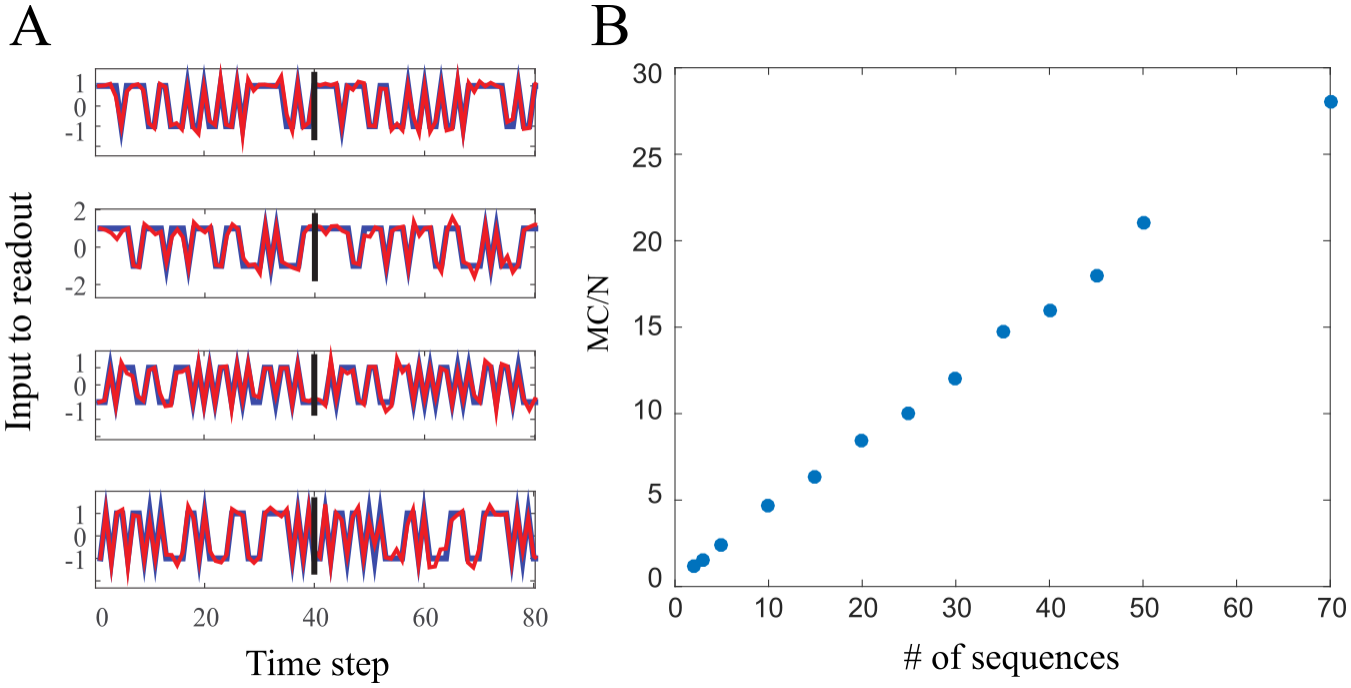
Parallel learning. (A) Learning 4 sequence (40 time step each) in parallel. Each panel corresponds to a different target sequence. Blue color represents the target sequence activity projected on the output weights **J**. Red color stands for the post learning noisy dynamic (*σ*_*noise*_ = 10^−4^) projection on the readout weights, **J**. Note that despite the noisy dynamics, the network is capable of reproducing perfectly the learned sequences. Noise causes small deviations from the desired activity in the network, which decays exponentially, leaving no trace on the output unit, *z*. (B) the memory capacity per neuron Vs. the number of sequences, *s*, one wishes to learn in parallel, such that each sequence is generated by cuing the network with an appropriate initial condition. For each number of sequences to learn in parallel, we looked for the maximal length of each sub-sequence, such that all *s* of them could be learned by a single output weight vector.

*Learning several sequences in parallel.* From a biological perspective, it seems common that a given network will be able to learn several sequences, for example several motor programs. Thus an important feature of the system is its ability to learn several sequences in parallel i.e. for a single set of learned weights, the system should be capable reproducing several different sequences. Distinct sequences will be generated upon cuing the network with appropriate initial conditions. We found that in our suggested learning scheme the network is capable in doing so. It is thus of interest to compare the network performance in this case, to the single sequence case. As an instructive example Fig 4A shows that a network with *N = 100* units is capable of learning 4 sequences in parallel (40 time steps each). After learning the network was cued, at each trial, with a different initial condition (each correspond to a different target sequence) and released to naturally evolve according to Eqs (1) and (2). Indeed the network exhibit a stable reproduction of the target sequences, over the output unit. Deviations from the desired activity are obviously observed, but these perturbations decay exponentially, leaving a perfect reproduction of the target sequence over the readout unit, *z*.

We now turn in quantifying the network performance in the case of learning, *s*, sequences in parallel. i.e. given an initial condition ${x_{0}}^{\mu }$, the network will produce the sequence ${\left\{z_{t}^{\mu }\left(n\right)\right\}}_{n=0}^{T^{\mu }}$. Where 1 ≤ *μ* ≤ *s* represent the *μ*^*th*^ sequence. Each sequence has its own training set ${\left\{x^{\mu }\left(n\right),z_{t}^{\mu }\left(n\right)\right\}}_{n=0}^{T^{\mu }}$. The training set in the parallel case is: $\cup_{\mu =1}^{s}\left({\left\{x^{\mu }\left(n\right),z_{t}^{\mu }\left(n\right)\right\}}_{n=0}^{T^{\mu }-1}\right)$, which is the union of all individual training sets together. Thus in order for the network to learn, *s*, sequences we should be able to solve the perceptron problem for this combined training set. In addition we will define the memory capacity as the maximal total length of all sequences together, i.e. $\max_{s,T^{\mu }}\left(\sum_{\mu =1}^{s}T^{\mu }\right)$. For simplicity we will examine the case where all sequences are of equal length, *T*^*μ*^ = *T*, ∀*μ*. In simulations we look for the maximal sequence length, *T*^*max*^, for every *s* (number of sequences to learn in parallel). Numeric results exhibit a dramatic effect (Fig 4B) relative to the single sequence case. For *s* ≲ 15, the maximal sub-sequence length slowly decreases to ∼40 time steps. From *s* > 15, it seems that the network could be loaded with many sub-sequences (we checked up to 70), hence the MC roughly grows linear with the number of sub-sequences. It is an interesting result since a naive thinking would predict that given a single sequence, one can divide it in an arbitrary manner to a number of individual sequences and the network will be capable of learning them. Hence, a naive approach will predict that parallel learning will not affect the MC, which clearly is not the case.

### Noise robustness

In the presence of noise the networks trajectory in phase space will have a probabilistic nature. Each point in phase space, obtained by Eq (3) will be smeared to a *N-1* ball of possible states. Hence noise robustness in the system stems from the finite margin of the perceptron problem. The quantity

$$\begin{array}{c} D\left(J\right)=\frac{1}{\left|J\right|}\min_{n}J\cdot x\left(n\right) \end{array}$$ (6)

Defined in [24] quantifies the difficulty level of the classification problem at hand. It is the worst projection from the set ${\left\{x(n)\right\}}_{n=1}^{T}$ on the hyperplane perpendicular to **J**. The best solution, i.e. with largest margin is obtained by maximizing the value of *D* over all possible weight vectors **J**:

$$\begin{array}{c} D_{max}=\max_{J}D\left(J\right) \end{array}$$ (7)

This is the margin, κ, obtained by the learning algorithm we used. The robustness to noise will be the order of magnitude of noise $\left(\sigma_{noise}^{2}\right)$ we can apply on each neuron, such that a learned sequence is still stable i.e. the hyperplane **J** classifies correctly the the set ${\left\{x_{noise}\left(n\right),z\left(n\right)\right\}}_{n=1}^{T}$, for many cycles before an error occurs. In order to quantify this we use Eq (1) to solve recursively for the activation pattern over the generator neurons, this time taking into account the noise term.

$$\begin{array}{ccc} x_{noise}\left(n\right) & = & W^{n}x\left(0\right)+\sum_{k=0}^{n-1}W^{k}Vz\left(n-k\right)+\sum_{k=0}^{n-1}W^{k}\eta \left(n-k\right)\\ & = & x(n)+R(n) \end{array}$$ (8)

where we denoted the noisy activity pattern by **x**_*noise*_. As expected, Eq (8) implies that the effect of noise is to drive the original activity pattern by $R\left(n\right)=\sum_{k=0}^{n-1}W^{k}\eta \left(n-k\right)$, which represent the accumulation of noise at the *n*^*th*^ time step. Thus in order for the output weights, **J**, to classify correctly the noisy dynamics we need to find *σ*_*noise*_ for which ∀*n*, ‖**x**(*n*) − **x**_*noise*_(*n*)‖ < κ, i.e. by Eq (8) we need to satisfy:

$$\begin{array}{c} ∥R(n)∥<\kappa \;\forall n \end{array}$$ (9)

Calculations show (S1 Text) that under the annealed approximation, the amount of noise that the network can tolerate is given by:

$$\begin{array}{c} \sigma_{noise}^{2}<\left(\frac{\kappa^{2}}{N}\right)\frac{1-N\sigma_{W}^{2}}{1-{\left(N\sigma_{W}^{2}\right)}^{n}} \end{array}$$ (10)

Where *σ*_*W*_ denotes the variance of an element in the connectivity matrix **W**, $\left(W_{ij}∼N\left(0,\sigma_{W}^{2}\right)\right)$. Note that in the large *N* limit *σ*_*W*_ is tightly related to |λ|–the maximal eigenvalue of **W**, through:

$$\begin{array}{c} \sigma_{W}^{2}=\frac{\lambda^{2}}{N} \end{array}$$ (11)

Plugging this relation in Eq (10) yields:

$$\begin{array}{c} \sigma_{noise}^{2}<\frac{\kappa^{2}}{N}\left(\frac{1-\lambda^{2}}{1-\lambda^{2n}}\right) \end{array}$$ (12)

$$\begin{array}{c} \lim_{n\to \infty }\left\{ \begin{array}{cc} \frac{\kappa^{2}}{N}\left[1-\lambda^{2}\right] & \lambda <1\\ 0 & \lambda >1 \end{array}\right. \end{array}$$ (13)

**Fig 5:**
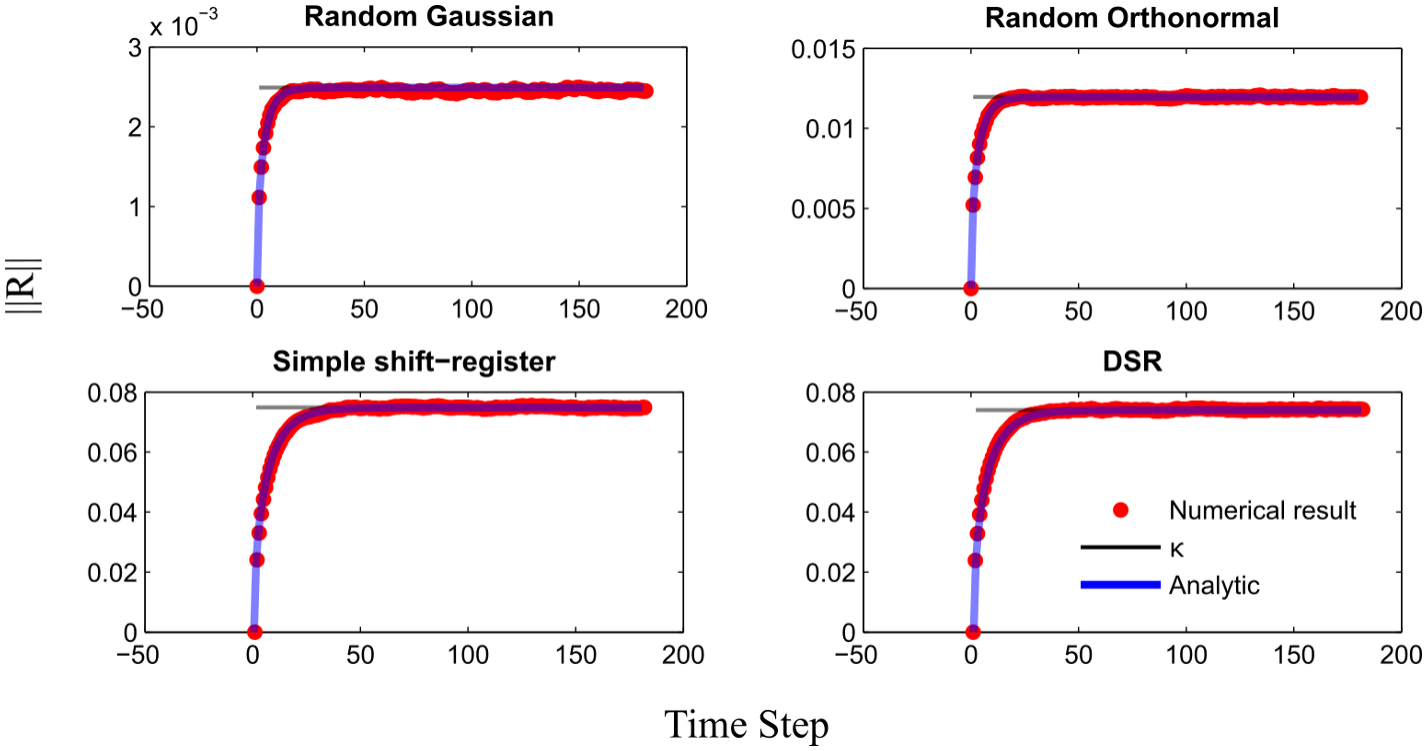
Noise robustness. Validity check for the analytic approximation of noise robustness in the system. Results are given as an average over the noise with fixed target sequence and connectivity. In each realization we let a *N* = 300, λ = 0.9 network learn a 60 time step random sequence. We then simulated the network trajectory for 3 cycles according to Eqs (1) and (2). Where *σ*_*noise*_ was chosen such it saturates the bound given by Eqs (13) and (15). We calculated and present ‖**R**(*n*)‖, at each time step. The red dashed curve is the analytic approximation, the blue curve is the averaged result from the simulation. We see that the analytic approximation indeed fits well the simulations.

This result is confirmed by numerical simulations in which we calculated ‖**R**(*n*)‖ for *σ*_*noise*_ that saturates the bound predicted by Eq (12). The average value of ‖**R**(*n*)‖ over the noise is compared to our prediction in Fig 5. On average results coincide, supporting the prediction for the scale of noise the network can tolerate. From Eq (13) we note that for λ > 1, the noise in the system grows exponentially resulting in an unstable system to noise perturbations. It further suggests that the robustness to noise depends both on the normalization of **W** and on κ. We can explicitly determine λ, but κ is given per certain realization of **W, V** and a specific target sequence {*z*_*t*_}. On the one hand, if we fix κ we get that increasing λ lowers the robustness to noise. But note that, as expected, simulations shows that κ is monotonically increasing function in λ (Fig 3D). This indicates that there exist an optimal λ, such that the robustness to noise of the system is ideal. This value will represent the counter balance between the ability to forget the errors and memorizing the desired sequence.

### Other weight matrices

So far our analysis focused on random connectivity, as we avoided from constraining the connectivity. In this section we will mention other classes of connectivity suggested for short term memory [25].

#### Shift-register networks

We will start by considering the simplest construction, the shift register. The simplest shift operator is given by *W*_*ij*_ = *λδ*_*i*, *j*+1_. A major drawback of this form is its extreme sensitivity to removal of a single neuron. A more robust, distributed architecture of the shift register operation is a fully connected network with

$$\begin{array}{c} W=\lambda \sum_{k=1}^{N-1}v^{\left(k+1\right)}v^{(k)T} \end{array}$$ (14)

where {**v**^*k*^} is an arbitrary set of *N* orthonormal vectors. Note that this architecture operates like the simple delay line since **W** **v**^*k*^ = λ **v**^*k*+1^. A clear advantage of these architectures is that they ensure the use of their *N* degrees of freedom for memory embedding. Indeed, simulation shows that for both the simple and distributed shift register, the memory capacity for λ = 0.999 roughly scales as *1.5 N* (S1 Fig). This is a surprising result considering that **W** is nilpotent matrix of order *N*. Hence feedback inputs from times earlier than *N* can’t interfere with current inputs. Nonetheless, given an initial condition, different feedback inputs leads to different, **x**, states of the network. Noise robustness calculations (S1 Text) suggest that in order for the solution to be stable, asymptotically the noise should satisfy

$$\begin{array}{c} \sigma_{noise}^{2}<\frac{\kappa^{2}}{N}\left[1-\lambda^{2}\right] \end{array}$$ (15)

note that asymptotically the noise has similar effect as for the random Gaussian case. Differences in robustness between the cases depends on κ, which is reminiscent of the different dynamics.

#### Random orthogonal network

A natural extension of the shift-register operation is to a network with **W** = λ **O**, where **O** is an *N* × *N* orthogonal matrix. In contrast to the shift-register, orthogonal **W** is full rank and inputs from times earlier than *N* can interfere with current inputs. Numerical simulations suggest that for matrices drawn from the Gaussian orthogonal ensemble the memory capacity per neuron for, λ = 0.999, pushes to Cover’s bound of *2N* (S1 Fig). Robustness to noise in this case is similar to the random generic case.

### Model generalization - Concrete examples

In this section we consider two tasks that our model, in its generalized form, can easily accomplish, without the need for any parameters fine tuning.

#### Tapping experiment

Here we adopt a sequence learning task (SRT) from [26]. The SRT is a four-choice reaction time task in which visual cues are linked to spatial-specific motor responses [27]. In one of its forms visual cues appear in any one of four possible positions arranged horizontally on a touch tablet. the responses are made by rapidly touching the cued location with a single finger. The cues are presented in a fixed, structured series of spatial locations; thus, unbeknown to the subjects, the cues introduce a sequence of lateral movements to be learned [28].

In order to use our model for this task we need to consider slight generalization (Methods), i.e. two output units are required to account for any of the four possible positions on the touch tablet. We will account different feedback loop weights for every output unit, and normalize them such that the total feedback is O(1). In the learning phase we will have to solve the perceptron problem twice, once for every output unit.

In [26] part of the subjects had to learn two different, 12 steps sequences, namely S12 (1-2-1-4-3-2-4-1-3-4-2-3) and R12 (3-2-4-1-3-1-2-3-4-2-1-4), where 1- stands for the left most key on the touch tablet, and 4—to right most. In order to transform these sequences to valid target sequences for our model we used the map in Table 1. Our model could easily learn this task, i.e. 20 neuron with wide scaling of λ managed to learn both sequences, in parallel, successfully.

**Table 1:** Map from output units state to key number on the touch-pad.

Output units state	key number
{-1,-1}	1
{1,-1}	2
{-1,1}	3
{1,1}	4

#### Learning to play a melody

Here we consider a task previously proposed in [13], where they used echo state architecture with 400 neurons to learn the melody of the “House of the rising sun”. This task forces us to use three binary output units, since the melody consists of eight different notes. We assign different states over the output units to different notes as shown in Table 2. The target sequence, taken from [13], when written in terms of notes is

$$\begin{array}{ccc} z_{t} & = & \{A,A,A,B,B♯,B♯,D♯,C♯,C♯,A,B♯,B♯,A^{\prime },A^{\prime },A^{\prime },A^{\prime },G^{\prime },D♯,C♯,D♯,\\ & & D♯,D♯,D♯,D♯,A^{\prime },A^{\prime },A^{\prime },A^{\prime },G^{\prime },G^{\prime },D♯,C♯,C♯,A,B♯,B♯,\\ & & A,A,A,A,G♯,G♯,G♯,A,A,A,A,A\} \end{array}$$ (16)

**Table 2:** Map from output units state to numerical values which represent notes, according to [13].

Output units state	Numerical value	Note
{-1,-1,-1}	-1	*G*♯
{1,-1,-1}	0	*A*
{1,1,-1}	2	*B*
{1,-1,1}	3	*B*♯
{-1,-1,1}	5	*C*♯
{-1,1,1}	7	*D*♯
{-1,1,-1}	12	*G*′
{1,1,1}	14	*A*′

Learning has been done similarly as in the previous example, this time learning three different output weights. We found that our network easily learns this task, i.e. with completely random connectivity, and for a wide range of normalization factors, λ. For example the minimum number of neurons (λ = 0.75) required for that task is 21, with κ ∼ 10^−3^. With 400 neurons (λ = 0.999) the margin increased by two order of magnitude, κ ∼ 3 ⋅ 10^−1^. This is another example to the ability of our model to generalize easily, accounting for multiple output units. Note that it also boosts its performance, relative to the single unit case, as in this example the memory capacity per neuron >2, which is impossible with single output unit.

## Discussion

We presented a simple solution to the stability problem in learning temporal sequences by recurrent networks. By considering a linear activation function for a recurrent neural network with feedback loop, we could cast the problem of learning a temporal sequence of actions over the output unit to a simple perceptron problem. Using our method we could get a perfect reproduction of a target sequence for many trials, even in the presence of noise. The robustness to noise was calculated in terms of the perceptron solution margin.

**Non-linear classification** This work only considered linear classification for simplicity. Allowing for non-linear classification, e.g. by the kernel method [29], one can potentially improve the performance of the network. Indeed, we managed to improve the memory capacity by roughly tenfold, using a radial basis kernel (Methods). In order to best exploit this method one should systematically search for the best kernel and an optimal procedure to determine its parameters values, which was out of scope in this work.

**Parallel learning** A dramatic effect has been observed when facing the task of learning several sequences in parallel. In our model it is possible to learn many sequences, such that the total number of actions the network can learn, substantially overcomes the length of the single sequence maximal length. While the core reason for this property remains mysterious for us, we would like to discuss a mathematical explanation and what it biologically infer. Mathematically the ability to learn sequence or sequences depends on the distribution in phase space of the training set. In our model, strong correlations are induced by the feedback loop, i.e. by the statistics of the target sequence. As mentioned in [20–22] the memory capacity, of the perceptron, monotonically increase with increased correlations in both input and output. Even though they considered different model, we believe this finding stands in one with the effect observed in parallel learning. In our model different sequences are correlated in some manner through the dynamics (**W**, **V**) and the similar statistics of the target sequence.

In the biological aspect, our finding suggest that it is economical to use a neural microcircuit for learning several short sequences rather than a long single sequence, which is a desirable property from a memory circuit.

**Other weight matrices** Our work focused on random connectivity, as we avoided from making assumptions on the internal structure. Nonetheless, major improvement in the network performance has been observed for other types of weight matrices. The special structure of these matrices better exploit the *N* degrees of freedom available to the network for memory embedding. This property also makes the memory capacity extensive. This finding should motivate future work that might consider learning procedures allowing for internal synapses modifications.

**Multiple output units** In this work we considered two examples of generalizing our model for multiple output units (two and three). Generally, the model will generalize to an arbitrary number of output units. But, since the output states available for the network are exponential in the number of output units, only small number of these are sufficient to produce a fairly rich output sequence. The performance for multiple output units haven’t been studied systematically in this work. Nonetheless, we note that considering multiple output units is beneficial for the network performance. e.g 21 neuron are sufficient to study a 48 step periodic sequence (melody of the “House of the rising sun”), while with a single output unit, a network with 20 neuron could maximally learn a 30 step periodic sequence. Note that in the case of multiple units the network is driven, by its own feedback, in various directions. That compared to the case of a single output unit, which only feeds back on a single vector, **V**. Thus multiple output units encourage the dynamic of the network to span larger volume in phase space, making the perceptron problem easier.

**Continuous time case** Extension of our model to a Continuous time representation is considered in S2 Text. Nonetheless, such an extension turned out yielding a major drawback. While in the discrete time case our method succeed in providing a robust solution, in the continuous time case it failed, as the margin approaches zero every time there is a jump in the target sequence. As a result, in the continuous case, the network was only able to reproduce the sequence with small jitters. It was numerically evident that the network is vulnerable to noise in the initial condition alone—hence it is vulnerable to noise in general. Changing the learning procedure, i.e. allowing modification in all synapses, internal and feedback connections, might help stabilizing the solution. Note that by taking this route the problem isn’t a simple perceptron problem any more, so a new learning rule should be obtained. One should note that modifying connections within the network, also changes the itinerary of the neural dynamic in phase space. This fact is what turns such an approach to a challenging one. Other works [15] used this approach, but as mentioned before, the network activity will eventually deviate from its target function outside the training window.

Timing is fundamental component for many of our day to day tasks. Yet, the neural mechanism underlying temporal processing remain unknown and controversial. It is not clear whether timing is dedicated to certain brain areas, or it is a general property, emerging from the neural activity. In our approach we used the dynamics of a recurrent neural network to implicitly represent time. That is, we encoded the timing of actions in the dynamics of the network.

From our results it is hard to be conclusive regarding this question. On the one hand, from the discrete time case it is evident, that indeed it is possible to encode time in a robust manner within the neural activity. On the other hand, in the continuous case we did face stability problem, which might only be a property of our mathematical solution.

In our mind, if it possible to robustly encode time in the discrete case, it should also be possible in the continuous case. As a consequence we do believe that this work support the claim in which timing is a general property of the brain, emerging from the neural activity.

## Methods

### The perceptron learning algorithm

In simulations we solved both the primal and dual form of the soft margin perceptron problem, as defined in [29, 30]. For learning multiple sequences in parallel we used the primal formulation, for the longest single sequence we used the dual formulation. The primal problem takes the following form

$$\begin{array}{cc} minimize:\; & \frac{1}{2}J\cdot J+\lambda \sum_{i=1}^{T}\xi_{i}\\ subject\;to:\; & \xi_{i}\geq 0\;\forall i\in 1,..,T\\ & z_{t}^{i}\left(J\cdot x_{i}+b\right)\geq 1-\xi_{i} \end{array}$$

Where **J** is the separating hyperplane and *ξ*_*i*_’s are slack variables, yielding *ξ*_*i*_ = 0 for patterns on the correct side of the margin. 0 < *ξ*_*i*_ < 1 for patterns in the margin and *ξ*_*i*_ > 1 for wrongly classified patterns. In this formulation choosing small values of λ will encourage a large margin, with possible not optimal performance on the training data, while large values of λ will encourage a solution that performs well on the training data. The advantage of solving the “soft” problem is that a solution that minimizes the objective function exists. We used λ = 10^48^ which effectively serves as λ → ∞, to encourage correct classification over the training data. The dual problem takes the following form

$$\begin{array}{cc} minimize:\; & \frac{1}{2}\sum_{i,j=1}^{T}\alpha_{i}\alpha_{j}z_{t}^{i}z_{t}^{j}\left(x_{i}\cdot x_{j}\right)-\sum_{i=1}^{T}\alpha_{i}\\ subject\;to:\; & 0\leq \alpha_{i}\leq \lambda \;\forall i\\ & \sum_{i}\alpha_{i}z_{t}^{i}=0 \end{array}$$

from the dual formulation the separating hypeplane is given by the support vectors

$$\begin{array}{c} J=\sum_{i=1}^{T}{\hat{\alpha }}_{i}z_{t}^{i}x_{i} \end{array}$$ (17)

the bias is calculated as a weighted average of the $\alpha_{i}^{\prime }s$, to deal with roundoff errors

$$\begin{array}{c} b=\sum_{i=1}^{T}{\hat{\alpha }}_{i}\left(z_{t}^{i}-J\cdot x_{i}\right)/\sum_{i}{\hat{\alpha }}_{i} \end{array}$$ (18)

Numerically we used the the matlab function *quadprog*, to solve both types of optimization problems. We set it with *interior-point-convex* algorithm and maximum number of iterations of 9000, to prevent it from terminating prematurely.

**Non-linear classification** Solving the dual problem generalizes easily for solving a non-linear classification problem by choosing an appropriate kernel [29], i.e substituting **x**_*i*_ ⋅ **x**_*j*_ with a general kernel *K*(**x**_*i*_, **x**_*j*_). Individual simulations with radial basis kernel, $K\left(x_{i},x_{j}\right)=\exp[-\frac{{\left|\right|x_{i}\;-\;x_{j}\left|\right|}^{2}}{2\sigma^{2}}]$, and *σ* set to typical distance between vectors, could increase the memory capacity by an order of magnitude (Not shown). This observation is based on single trials and not studied systematically.

### Simulations technique

The connectivity matrix, **W**, was constructed such that its largest eigenvalue is of particular value λ. To do so we first draw a random matrix with elements ${\tilde{W}}_{ij}∼N\left(0,\frac{\lambda^{2}}{N}\right)$, and applied normalization such that $W=\frac{\lambda }{\lambda_{max}}\tilde{W}$. Where λ_*max*_ is the largest eigenvalue, in absolute value of $\tilde{W}$. Every element in the feedback weight vector, *V*_*i*_, was drawn from a standard normal distribution and normalized such that ‖**V**‖ = 1. In each trial of the simulation we where interested in learning a specific binary sequence {*z*_*t*_} of length *T*, such that, *z*_*t*_(*t*) = ±1 with probability $\frac{1}{2}$. For each setup of random connections **W**, **V** we let the network learn various random sequences {*z*_*t*_} of different length *T*. After learning the output weight vector **J**, we have simulated the network dynamic with Eqs (1) and (2) for 5 cycles (e.g. for target sequence *T*, we have simulated the network dynamics for 5*T* time steps). Eventually we compared the simulated output versus the target sequence {*z*_*t*_} counting for erroneous actions. For each sequence length we averaged the error over 300 repetitions of different random sequences. In addition we have done so for a given network setup over different normalization of **W**, i.e. different values of λ, note that that we have trained each specific random pattern over all different normalization of **W**. Following this procedure we have constructed the memory curve for a given network of size *N*, see S1 Fig for example. From such figure we extracted the memory capacity (MC) for each normalization of **W**, we have done so by taking the point on which the derivative of the curve is largest. Doing so for different realization of network setups we have plotted the memory capacity normalized by the network size (*N*) for different normalization of **W**, as can be seen in Fig 3.

**Several sequences in parallel** In order to construct Fig 4 we used Simulations of equal length sub-sequences. Given a number (denote by *s*) of sequences we wish to learn in parallel. We look for the maximal length, $T_{s}^{max}$, for which we can learn this set of *s* sequences. Sequences are again binary with equal probability to be in each state. The memory capacity for a set of *s* sequences is just $sT_{s}^{max}$.

**Multiple output units** A generalization of the model is to consider an arbitrary number, *l*, of output units, *z*_*i*_, *i* = 1, 2, …, *l*, generally satisfying *l* ≪ *N*. Each output unit has its own feedback loop **V**_*i*_, keeping the total feedback O(1), requires $\left||V_{i}|\right|=½\frac{1}{\sqrt{l}}$. In the learning phase the perceptron problem is solved for each output unit separately, i.e. finding the best hyperplane, **J**_*i*_ for each unit. Note that the margin in this case is defined by $\underset{i}{\min\kappa_{i}}$, i.e. the minimal margin from all of the *l* perceptron problem solved.

## Supporting information

S1 Text The discrete time case. This section includes noise robustness calculations for several weight matrices.
(PDF)

S2 Text The continuous time case. In this section we generalize our model to continuous time.
(PDF)

S1 Fig Memory curves for the Delay-line and random orthonormal connectivity. (TIF)

S2 Fig Example for learning a continuous sequence. (TIF)

S3 Fig The continuous solution dynamics. (TIF)

S4 Fig Memory capacity for the continuous case. (TIF)

S5 Fig Example of the continuous time learning algorithm. (TIF)

## References

[1] Reiss R. A theory of resonance networks Neural theory. 1964;.

[2] Harmon LD. Neuromimes: action of a reciprocally inhibitory pair. Science. 1964;146(3649):1323–1325. doi: 10.1126/science.146.3649.1323 14207464 10.1126/science.146.3649.1323

[3] Wilson DM, Waldron I. Models for the generation of the motor output pattern in flying locusts. Proceedings of the IEEE. 1968;56(6):1058–1064. doi: 10.1109/PROC.1968.6457

[4] Kling U, Székely G. Simulation of rhythmic nervous activities. Kybernetik. 1968;5(3):89–103. doi: 10.1007/BF00288899 5728516 10.1007/BF00288899

[5] Kleinfeld D, Sompolinsky H. Associative neural network model for the generation of temporal patterns. Theory and application to central pattern generators. Biophysical Journal. 1988;54(6):1039 doi: 10.1016/S0006-3495(88)83041-8 3233265 10.1016/S0006-3495(88)83041-8 PMC1330416

[6] Kleinfeld D, Sompolinsky H. Associative network models for central pattern generators. Methods in neuronal modeling. 1989;p. 195–246.

[7] Hebb DO. The organization of behavior. New York: Wiley; 1949.

[8] Jordan MI. Serial order: A parallel distributed processing approach. Advances in psychology. 1997;121:471–495. doi: 10.1016/S0166-4115(97)80111-2

[9] Elman JL. Finding structure in time. Cognitive science. 1990;14(2):179–211. doi: 10.1207/s15516709cog1402_1

[10] Horton JC, Adams DL. The cortical column: a structure without a function. Philosophical Transactions of the Royal Society B: Biological Sciences. 2005;360(1456):837–862. doi: 10.1098/rstb.2005.1623 10.1098/rstb.2005.1623 PMC1569491 15937015

[11] Maass W, Joshi P, Sontag ED. Computational aspects of feedback in neural circuits. PLoS Comput Biol. 2007;3(1):e165 doi: 10.1371/journal.pcbi.0020165 17238280 10.1371/journal.pcbi.0020165 PMC1779299

[12] Dominey PF. Complex sensory-motor sequence learning based on recurrent state representation and reinforcement learning. Biological cybernetics. 1995;73(3):265–274. doi: 10.1007/BF00201428 7548314

[13] Jaeger H. The “echo state” approach to analysing and training recurrent neural networks-with an erratum note. Bonn, Germany: German National Research Center for Information Technology GMD Technical Report. 2001;148:34.

[14] Maass W, Natschläger T, Markram H. Real-time computing without stable states: A new framework for neural computation based on perturbations. Neural computation. 2002;14(11):2531–2560. doi: 10.1162/089976602760407955 12433288 10.1162/089976602760407955

[15] Sussillo D, Abbott LF. Generating coherent patterns of activity from chaotic neural networks. Neuron. 2009;63(4):544–557. doi: 10.1016/j.neuron.2009.07.018 19709635 10.1016/j.neuron.2009.07.018 PMC2756108

[16] Jaeger H, Haas H. Harnessing nonlinearity: Predicting chaotic systems and saving energy in wireless communication. Science. 2004;304(5667):78–80. doi: 10.1126/science.1091277 15064413 10.1126/science.1091277

[17] Schiller UD, Steil JJ. Analyzing the weight dynamics of recurrent learning algorithms. Neurocomputing. 2005;63:5–23. doi: 10.1016/j.neucom.2004.04.006

[18] Rosenblatt F. The perceptron: A probabilistic model for information storage and organization in the brain. Psychological review. 1958;65(6):386 doi: 10.1037/h0042519 13602029 10.1037/h0042519

[19] Rosenblatt F. Principles of neurodynamics. 1962;.

[20] Bressloff P, Taylor J. Temporal sequence storage capacity of time-summating neural networks. Journal of Physics A: Mathematical and General. 1992;25(4):833 doi: 10.1088/0305-4470/25/4/020

[21] Bressloff P, Taylor JG. Perceptron-like learning in time-summating neural networks. Journal of Physics A: Mathematical and General. 1992;25(16):4373 doi: 10.1088/0305-4470/25/16/014

[22] Clopath C, Nadal JP, Brunel N. Storage of correlated patterns in standard and bistable Purkinje cell models. PLoS Comput Biol. 2012;8(4):e1002448 doi: 10.1371/journal.pcbi.1002448 22570592 10.1371/journal.pcbi.1002448 PMC3343114

[23] Girko V. Circular law. Theory of Probability & Its Applications. 1985;29(4):694–706. doi: 10.1137/1129095

[24] Hertz J, Krogh A, Palmer RG, Horner H. Introduction to the Theory of Neural Computation. Physics Today. 2008;44(12):70–70. doi: 10.1063/1.2810360

[25] White OL, Lee DD, Sompolinsky H. Short-term memory in orthogonal neural networks. Physical review letters. 2004;92(14):148102 doi: 10.1103/PhysRevLett.92.148102 15089576 10.1103/PhysRevLett.92.148102

[26] Adini Y, Bonneh YS, Komm S, Deutsch L, Israeli D. The time course and characteristics of procedural learning in schizophrenia patients and healthy individuals. Frontiers in human neuroscience. 2015;9:475 doi: 10.3389/fnhum.2015.00475 26379536 10.3389/fnhum.2015.00475 PMC4555022

[27] Nissen MJ, Bullemer P. Attentional requirements of learning: Evidence from performance measures. Cognitive psychology. 1987;19(1):1–32. doi: 10.1016/0010-0285(87)90002-8

[28] Richard MV, Clegg BA, Seger CA. Implicit motor sequence learning is not represented purely in response locations. The Quarterly Journal of Experimental Psychology. 2009;62(8):1516–1522. doi: 10.1080/17470210902732130 19283555 10.1080/17470210902732130

[29] Cortes C, Vapnik V. Support-vector networks. Machine learning. 1995;20(3):273–297. doi: 10.1007/BF00994018

[30] Press WH, Teukolsky SA, Vetterling WT, Flannery BP. Numerical recipes in C. vol. 2 Cambridge Univ Press; 1982.

