# Supplementary material for: Stabilizing patterns in time: Neural network approach
Source: PLoS Comput Biol. 2017 Dec 12;13(12):e1005861. doi: 10.1371/journal.pcbi.1005861 (PMC5741269; doi:10.1371/journal.pcbi.1005861)
Supplement: S1 Text — This section includes noise robustness calculations for several weight matrices. (PDF) [file pcbi.1005861.s001.pdf]

S1 - Text

Noise robustness calculation

We would like to estimate the robustness to noise of several network architectures. For all cases we will have to calculate the following:

$$\langle||\mathbf{R}\left(t\right)||^{2}\rangle = \left\langle \left(\sum_{k=0}^{t-1} \mathbf{W}^{k}\eta\left(T-t\right)\right)^{2}\right\rangle \tag{S1}$$

Calculation depends on the particular structure of the connectivity matrix, in addition note that since our interest is in periodic sequences, the length of the sequence is not important and require stability for an arbitrary number of cycles. Since we restrict ourselves to cases in which the largest eigen/singular value is smaller than one, we expect the noise to saturate. In the following we will consider direct calculations for the shift-register and random Gaussian cases.

Shift register

Simple shift The shift register construction guarantees the use of N degrees of freedom available for network. A simple construction will be of the form $W_{ij} = \lambda\delta_{i,j-1}$. Calculation of Eq.(S1) is straight forward in this case since

$$\left(\mathbf{W}^{t}\right)_{ij} = \begin{cases} \lambda^{t}\delta_{i,j-t-1} & t < N \\ 0 & t \geq N \end{cases}$$

therefore

$$\begin{aligned} \langle\|\mathbf{R}\left(t\right)\|^{2}_{(shift)}\rangle &= \sum_{i=1}^{N}\left\langle \left(\sum_{k=0}^{t-1}\sum_{n=1}^{N}\lambda^{k}\delta_{i,n-k-1}\eta_{n}\left(t-k\right)\right)^{2}\right\rangle \\ &= \sum_{i=1}^{N}\sum_{l,k=0}^{t-1}\sum_{n,m=1}^{N}\lambda^{k+l}\delta_{i,n-k-1}\delta_{i,m-l-1}\langle\eta_{i}\left(t-k\right)\eta_{i}\left(t-l\right)\rangle \\ &= \sum_{i}^{N}\sum_{k=0}^{\min(t-i,N-i)}\lambda^{2k}\sigma_{\eta}^{2} \\ &= \sigma_{\eta}^{2}\begin{cases} \frac{N(1-\lambda^{2})+\left(\lambda^{2n}-1\right)\lambda^{-2n+2t+2}}{\left(\lambda^{2}-1\right)^{2}}, & t < N \\ \frac{\lambda^{2}(\lambda^{2N}-1)+N(1-\lambda^{2})}{(\lambda^{2}-1)^{2}}, & t \geq N \end{cases} \end{aligned} \tag{S2}$$

Asymptotically we note that the accumulated noise grows linearly with the network size $\sim \frac{N}{1-\lambda^{2}}$, and could not be compensated with an appropriate normalization of λ. We verified this result with

simulations presented in Fig. 5 in the main text.

Distributed shift register The structure of the simple shift is extremely sensitive for perturbation as single neuron removal. A more robust architecture is the distributed shift register (DSR) operation, which is a fully connected network with

$$\mathbf{W}_{(DSR)} = \lambda \sum_{k=1}^{N-1} \mathbf{v}^{(k+1)} \mathbf{v}^{(k)T}$$

where $\{\mathbf{v}^k\}$ is a set of N orthonormal vectors. First we note that

$$\mathbf{W}^t = \lambda^t \sum_{k=t-1}^{N-1} \mathbf{v}^{(k+1)} \mathbf{v}^{(k+t-1)T}$$

and for $t \geq N$, $\mathbf{W}^t = 0$, hence after N time steps the accumulation of noise should saturate. If we further assume that $v_i^k \sim N^{-1/2} \mathcal{N}(0,1)$, we could calculate the effect of the noise at every time step by

$$\begin{aligned}
\langle \|\mathbf{R}(t)\|^2_{(DSR)} \rangle &= \left(\sum_{\nu=0}^{\min(t-1,N-1)} \langle \mathbf{W}^\nu \eta(t-\nu) \rangle \right)^2 \\
&= \sum_{\nu,\mu=1}^{\min(t-1,N-1)} \sum_{k,l=t-1}^{N-1} \sum_{i,n,m=1}^{N} \frac{\lambda^{\nu+\mu}}{N^2} \langle v_i^{k+1} v_m^k v_i^{l+1} v_n^l \eta_n(t-\nu) \eta_m(t-\mu) \rangle \\
&= \sum_{\nu=0}^{\min(t-1,N-1)} \sum_{i,n=1}^{N-1} \sum_{k,l=t-1}^{N-1} \frac{\lambda^{2\nu} \sigma_\eta^2}{N^2} \langle v_i^{k+1} v_n^k v_i^{l+1} v_n^l \rangle \\
&= \sum_{\nu=0}^{\min(t-1,N-1)} (N-2-\nu) \cdot \lambda^{2\nu} \sigma_\eta^2 \\
&= \sigma_\eta^2 \begin{cases} \frac{\lambda^2 + N(\lambda^2-1)\left(\lambda^{2t}-1\right) + \left[t+2-\lambda^2(t+1)\right]\lambda^{2t} - 2}{(\lambda^2-1)^2}, & t < N \\ \frac{-\lambda^2 + \lambda^{2N+2} - 2\lambda^{2N} + N(\lambda^2-1) + 2}{(\lambda^2-1)^2}, & t \geq N \end{cases}
\end{aligned} \tag{S3}$$

as expected, the DSR accumulated noise behaviour shares similar asymptotic as the simple shift and the random Gaussian case, i.e. $\sim \frac{N}{1-\lambda^2}$.

Random Gaussian Recall that in this case $W_{ij} \sim \mathcal{N}(0, \sigma_W^2)$, with $\sigma_W = \frac{\lambda}{\sqrt{N}}$. Now note that $(W^k)_{ij}$ is a sum over N^{k-1} terms, were each term is multiplication of k normally distributed variables W_{ij}. For simplicity we can solve it under the annealed approximation, where at each time step we draw a new random matrix, in that way we won't have to deal with correlation arises

from matrix multiplication

$$\begin{aligned}\langle R_i^2(t)\rangle &= \left(\sum_{k=0}^{t-1}\left(\mathbf{W}^k\right)_{ij}\eta_j(n-k)\right)^2\\ &\approx \sigma_{noise}^2\left(\sum_{k=0}^{t-1}(N\sigma_W^2)^k\right)^2\\ &= \sigma_{noise}^2\left(\frac{\left(N\sigma_W^2\right)^t-1}{N\sigma_W^2-1}\right)^2\end{aligned} \tag{S4}$$

from Eq. (S4) one can find the overall noise effect by:

$$\begin{aligned}\left\langle\left\|\mathbf{R}^2(t)\right\|\right\rangle &\approx N\cdot\langle R_i^2(t)\rangle\\ &= N\sigma_{noise}^2\left(\frac{\left(N\sigma_W^2\right)^t-1}{N\sigma_W^2-1}\right)^2\end{aligned} \tag{S5}$$

Writing Eq. (S5) explicitly in terms of N and λ yields:

$$\left\langle\left\|\mathbf{R}^2(t)\right\|\right\rangle = N\sigma_{noise}^2\left(\frac{\lambda^{2t}-1}{\lambda^2-1}\right) \tag{S6}$$

Note that the random Gaussian case slightly differs in its asymptotic, compared to the shift-register. Here $\langle\mathbf{R}^2\rangle\sim\frac{N}{1-\lambda^2}$, i.e. we gain slightly more robustness by increasing λ. To get the result in the main text note that since we demanded $\|\mathbf{R}(n)\|<\kappa$, we can easily use Eq. (S6) to get a bound on σ_{noise}:

$$\sigma_{noise}^2<\frac{\kappa^2}{N}\left(\frac{1-\lambda^2}{1-\lambda^{2n}}\right) \tag{S7}$$

Noise Simulations In order to check the validity of our result on the noise bound, we have constructed a simple simulation in which we calculated $\|\mathbf{R}(n)\|$ at each step. We used a network with $N=100$ and $\lambda=0.9$, and constructed $\mathbf{W}$ and $\mathbf{V}$ as described in Methods. Given an arbitrary target sequence we found the initial condition $\mathbf{x}_0$, and simulated the following equations:

$$\begin{aligned}\mathbf{x}(n+1) &= \mathbf{W}\mathbf{x}(n)+\mathbf{V}z_t(n) && ;\mathbf{x}(0)=\mathbf{x}_{noise}(0)=\mathbf{x}_0\\ \mathbf{x}_{noise}(n+1) &= \mathbf{W}\mathbf{x}_{noise}(n)+\mathbf{V}z_t(n)+\eta(n) && ;\eta_i\sim\mathcal{N}\left(0,\sigma_{noise}^2\right)\end{aligned}$$

Where at each trial we set σ_{noise} to saturate the margin, such that $\sqrt{\langle\mathbf{R}^2\rangle}=\kappa$. We then calculated and plotted the reminder per unit, i.e. $\frac{\|\mathbf{R}(n)\|}{N}=\frac{1}{N}\|\mathbf{x}(n)-\mathbf{x}_{noise}(n)\|$, and the approximated reminder per unit (given by Eq. S2,S3,S5). We used the same target sequence for all connectivity types, and averaged over the noise, results are given in Fig. 5 at main text. Indeed it seems that simulations results coincides with our approximation.
