# Supplementary material for: Stabilizing patterns in time: Neural network approach
Source: PLoS Comput Biol. 2017 Dec 12;13(12):e1005861. doi: 10.1371/journal.pcbi.1005861 (PMC5741269; doi:10.1371/journal.pcbi.1005861)
Supplement: S2 Text — In this section we generalize our model to continuous time. (PDF) [file pcbi.1005861.s002.pdf]

S2 - Text

The Continuous Time Case

Model Details Here we explore a natural extension of our model, i.e. we would like to think on the network dynamic as continuous, or rate model. Therefore a natural generalization for discrete time model is:

$$\tau_x \frac{\mathrm{d}\mathbf{x}\left(\mathrm{t}\right)}{\mathrm{dt}} = -\mathbf{x}\left(t\right) + \mathbf{W}\mathbf{x}\left(t\right) + \mathbf{V}z\left(t\right) \tag{S8}$$

$$z\left(t\right) = \mathrm{sign}\left(\mathbf{J}\cdot\mathbf{x}\left(t\right)\right) \tag{S9}$$

Where sign$(x) = -1$ for $x \leq 0$, τ_x is the time scale of the generating neurons and $t \in [0, \infty)$, is a continuous variable representing time . We wish to examine whether the network is capable of learning a continuous target sequence, $z_t\left(t\right)$. As in the discrete case, we would like the network to reproduce the target sequence upon cuing it with the appropriate initial condition. In addition we would like the network to keep reproducing the target sequence periodically.

We chose to model the target sequence as a two-state ($z_t = \pm 1$) symmetric Markov process. We denote by τ_z^{-1} the process parameter, that reflects the rate in which the sequence changes state. With these assumptions we get that $\langle z_t\left(t\right)\rangle$=0, averaged over time, resembling the discrete case target sequence.

As in the discrete case, given a target sequence, $z_t\left(t\right)$, one can find the solution to Eq. (S8), where the initial condition is given from the periodicity constraint $\mathbf{x}\left(T\right) = \mathbf{x}\left(0\right)$:

$$\mathbf{x}\left(t\right) = \exp\left\{\left(\mathbf{W}-\mathbf{I}\right)\frac{t}{\tau_x}\right\}\mathbf{x}_0 + \tau_x^{-1}\left(\int_0^t \exp\left\{\left(\mathbf{W}-\mathbf{I}\right)\frac{t-s}{\tau_x}\right\}\mathbf{V}z_t\left(s\right)\mathrm{ds}\right) \tag{S9}$$

$$\mathbf{x}_0 = \tau_x^{-1}\left(\mathbf{I}-\exp\left\{\left(\mathbf{W}-\mathbf{I}\right)\frac{T}{\tau_x}\right\}\right)^{-1}\left(\int_0^T \exp\left\{\left(\mathbf{W}-\mathbf{I}\right)\frac{T-s}{\tau_x}\right\}\mathbf{V}z_t\left(s\right)\mathrm{ds}\right)$$

Eq. (S9), together with $z_t\left(t\right)$, forms an infinite training set. In order to incorporate the same learning method as in the discrete case, one should form a finite training set, from which we could infer a proper set of $\mathbf{J}$ weights. Finding a method to construct such a set, from which the network could generalize and classify the entire sequence correctly is the crux of the matter.

Our simulations show that finding such a solution is possible. S2 Fig is an example of a stable solution. The network is capable of reproducing the desired sequence periodically, with only slight jitters in both output unit and activity within the network. S2 FigC shows explicitly the *projected error*, $\mathbf{R}\left(n\right)\cdot\mathbf{J}$ at each time step. We note that most of the time the projected error is kept small, indicating that small perturbation rapidly decay. Larger error occurs in times the readout unit

produces erroneous output. In the case of a successful learning, this error decays exponentially and does not induce future errors.

Constructing The Training Set In order to find an appropriate training set we rely on an iterative method of re-sampling and learning, where we limit the number of iterations to three. Obviously, if the target sequence is learnable, then there exist a sampling set of the dynamics, one from which we can infer an appropriate set of $\mathbf{J}$ weights. Naturally a computational problem arises, as there are infinitely many possibilities to sample a single sequence. The challenge is thus; constructing a sampling algorithm which will be computationally reasonable and also reliable.

The sampling method we propose is as follows. First, we identify the transition points in the target sequence as key points. Therefore we start by sampling them, note that on average we have T/τ_z switching points in a sequence. In addition, we uniformly sample the sequence in τ_s^{-1} frequency, which fulfills $\tau_z^{-1} < \tau_s^{-1} < \tau_x^{-1}$. This choice reflects the fact, that in order for neuron with time constant τ_x be able to track a target sequence, which is switching states in τ_z^{-1} frequency, the following must hold $\tau_x < \tau_z$. Note that sampling the sequence in τ_s^{-1} frequency will provide us another T/τ_s points for the training set. Combining the set of points from the two sampling methods we form a finite training set $\{\mathbf{x}_{sample}(t_i), z_t(t_i)\}_{i=1}^{T\left(\tau_z^{-1}+\tau_s^{-1}\right)}$. We can now try and solve the perceptron problem for this set. In case the learning didn't converge we re-sample the sequence in points where the sequence wasn't classified correctly, adding these points to the previous points we update our training set. We can now follow this procedure until the solution converges, in our simulations we restricted the number of iterations to three, mostly from computational reasons.

The Solution Dynamics We first give a heuristic description of the solution dynamics, in terms of it's margin, κ. Therefore we start by plotting the averaged κ dynamics for a specific target sequence. That is, we use the same target sequence but change $\mathbf{W}$ and $\mathbf{V}$ in each realization. The results indicate (S3 Fig) that $\kappa \to 0$, every time the target sequence switches state. Hence the network could only hope producing the target sequence with small jitters. Thinking of trying to classify a continuous trajectory in phase space (i.e. $\mathbf{x}(t)$ trajectory), indeed reveals this difficulty. If we denote the time in which the target sequence changed state by t_j, we know from continuity that $\lim_{\Delta t\to 0}[\mathbf{x}(t_j+\Delta t)-\mathbf{x}(t_j-\Delta t)]=0$, hence for any weights, $\kappa \to 0$ at switching points of the target sequence. We now turn turn on quantifying it's performance.

Quantifying The Memory Capacity We quantify the MC as the longest sequence which the network is capable of learning (denoted by T^*). Since the nature of the target sequence is defined by its switching rate parameter τ_z^{-1}, an informative quantity for the MC is the dimensionless variable T^*/τ_z. This variable represents the expected, maximal number of switches the target sequence can have, and still be learnable. Results (S4 Fig) show that there exist an optimal ratio (τ_z/τ_x), for which the MC is maximized. Intuitively one may assume that an easy sequence for the network to

learn is when the typical time scale of the sequence resembles the typical time scale of the network. Hence from this numerical result we might estimate the effective time scale $\left(\tau_x^{eff}\right)$ of the network.

In the slow sequence dynamics, where the target sequence is switching states at a frequency much slower than the effective time scale of the network . The shape of the memory curve can be fairly estimated via statistical consideration alone.
