# Supplementary figures and images for: Stabilizing patterns in time: Neural network approach
Source: PLoS Comput Biol. 2017 Dec 12;13(12):e1005861. doi: 10.1371/journal.pcbi.1005861 (PMC5741269; doi:10.1371/journal.pcbi.1005861)

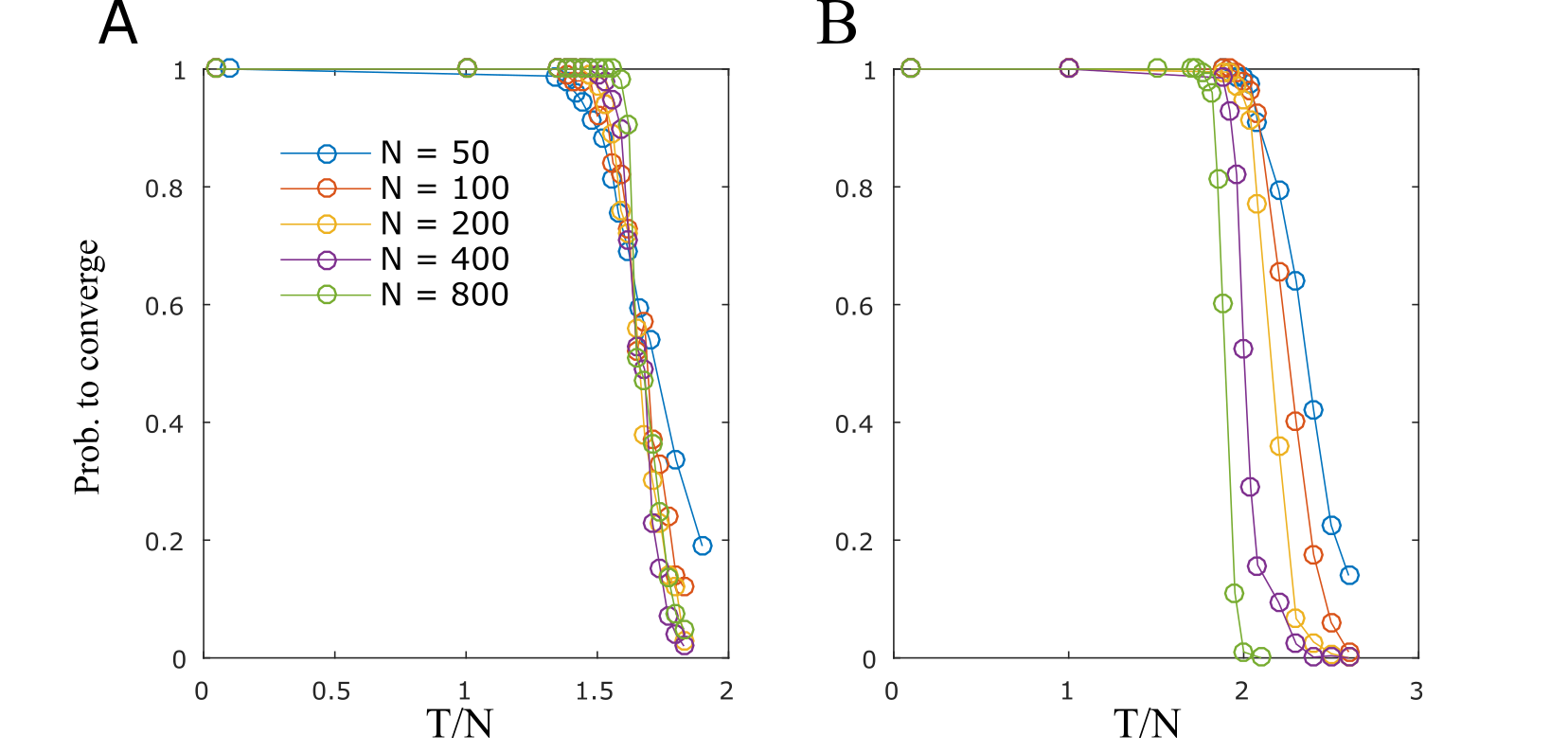

Supplement: S1 Fig — (TIF) [file pcbi.1005861.s003.tif]

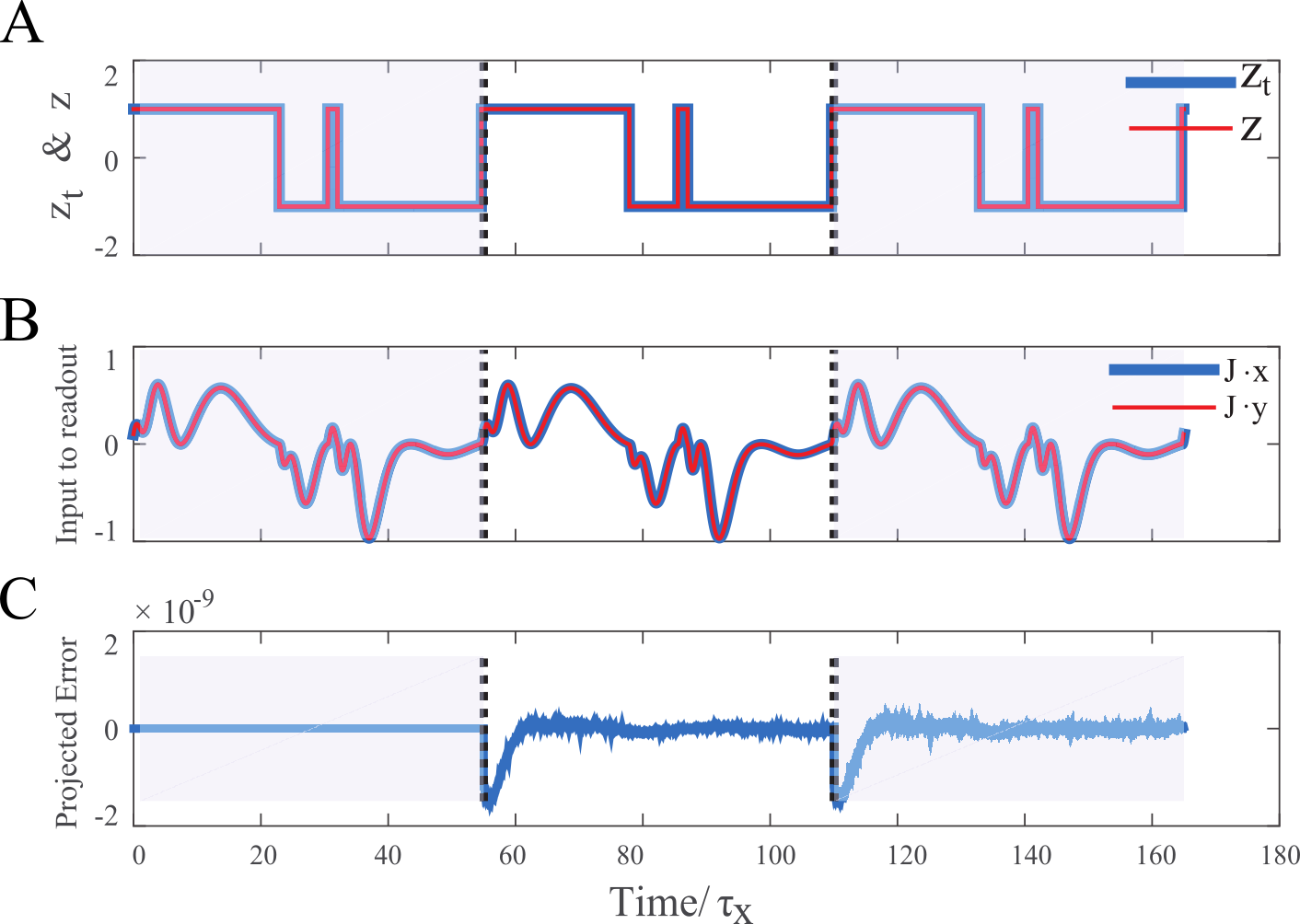

Supplement: S2 Fig — (TIF) [file pcbi.1005861.s004.tif]

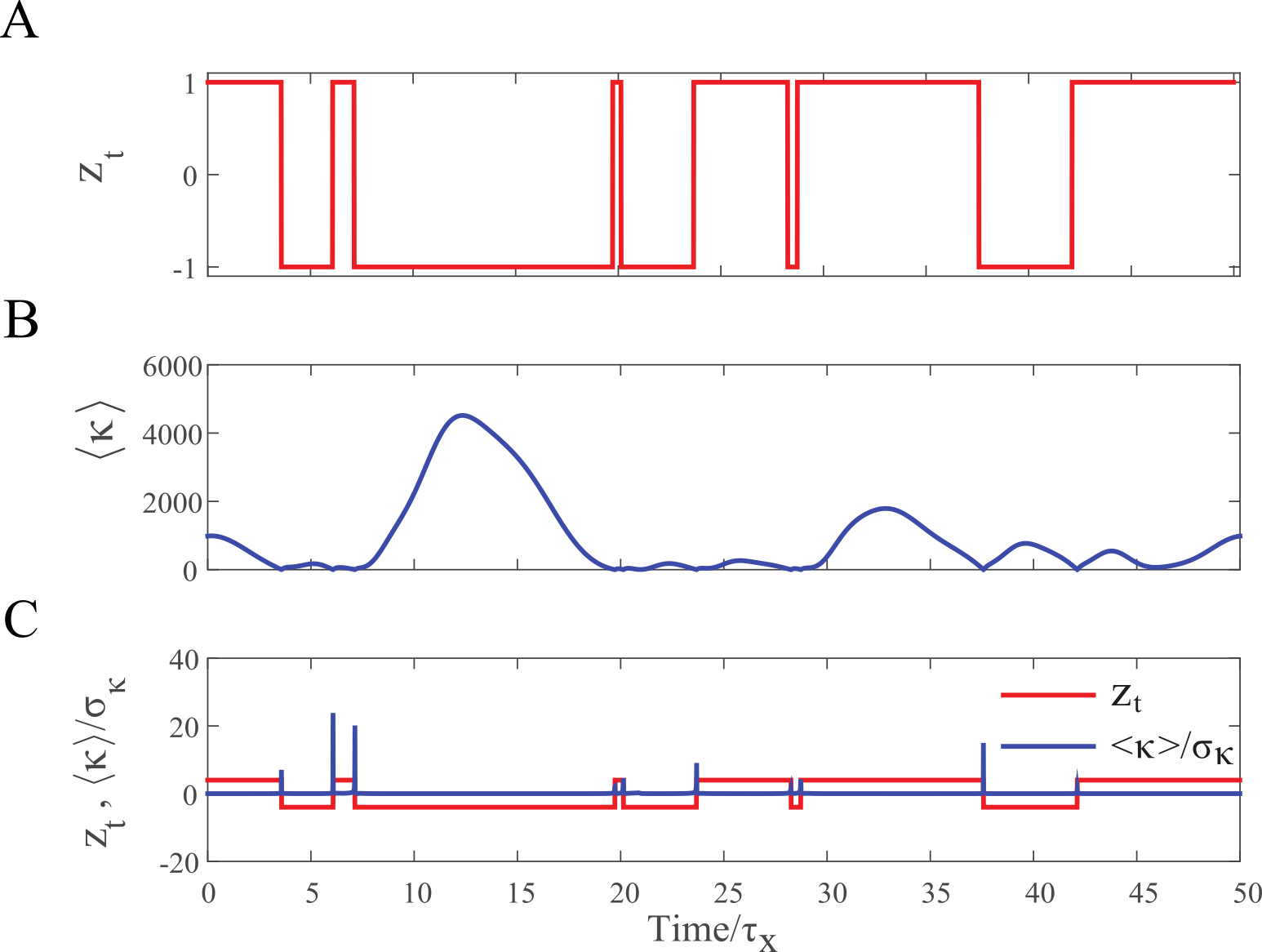

Supplement: S3 Fig — (TIF) [file pcbi.1005861.s005.tif]

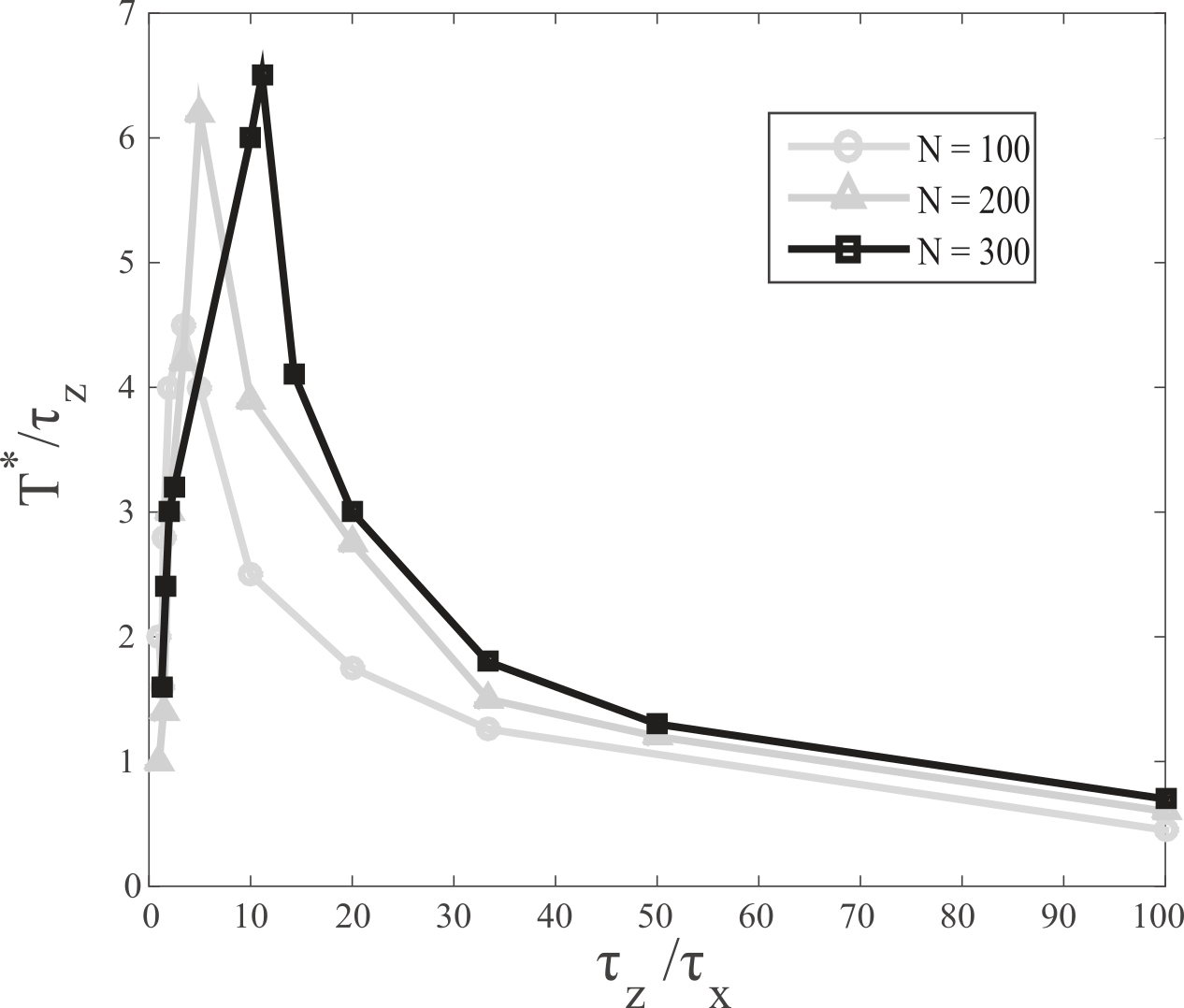

Supplement: S4 Fig — (TIF) [file pcbi.1005861.s006.tif]

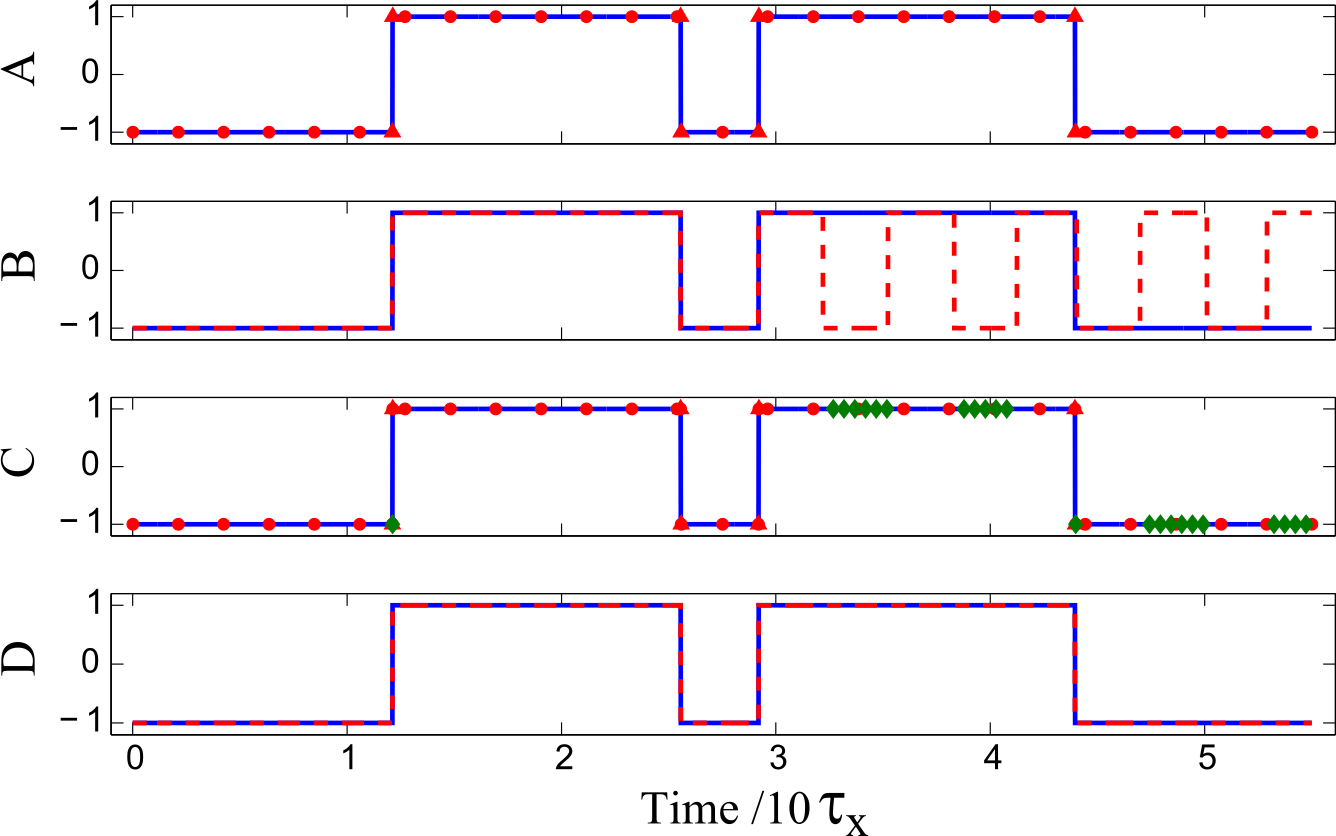

Supplement: S5 Fig — (TIF) [file pcbi.1005861.s007.tif]
